# Lessons From Transcriptome Analysis of Autoimmune Diseases

**DOI:** 10.3389/fimmu.2022.857269

**Published:** 2022-05-18

**Authors:** Yasuo Nagafuchi, Haruyuki Yanaoka, Keishi Fujio

**Affiliations:** ^1^ Department of Allergy and Rheumatology, Graduate School of Medicine, The University of Tokyo, Tokyo, Japan; ^2^ Department of Functional Genomics and Immunological Diseases, Graduate School of Medicine, The University of Tokyo, Tokyo, Japan; ^3^ Immuno-Rheumatology Center, St. Luke’s International Hospital, St. Luke’s International University, Tokyo, Japan

**Keywords:** transcriptome, eQTL, autoimmune disease, immune cell, monocytes, macrophages, systemic lupus erythematosus, rheumatoid arthritis

## Abstract

Various immune cell types, including monocytes, macrophages, and adaptive immune T and B cells, play major roles in inflammation in systemic autoimmune diseases. However, the precise contribution of these cells to autoimmunity remains elusive. Transcriptome analysis has added a new dimension to biology and medicine. It enables us to observe the dynamics of gene expression in different cell types in patients with diverse diseases as well as in healthy individuals, which cannot be achieved with genomic information alone. In this review, we summarize how transcriptome analysis has improved our understanding of the pathological roles of immune cells in autoimmune diseases with a focus on the ImmuNexUT database we reported. We will also discuss the common experimental and analytical design of transcriptome analyses. Recently, single-cell RNA-seq analysis has provided atlases of infiltrating immune cells, such as pro-inflammatory monocytes and macrophages, peripheral helper T cells, and age or autoimmune-associated B cells in various autoimmune disease lesions. With the integration of genomic data, expression quantitative trait locus (eQTL) analysis can help identify candidate causal genes and immune cells. Finally, we also mention how the information obtained from these analyses can be used practically to predict patient prognosis.

## Introduction

Autoimmune reactions and chronic inflammation are hallmarks of systemic autoimmune diseases or rheumatic diseases. The presence of autoantibodies and autoreactive T and B cells in these diseases indicates that the adaptive immune system is critical for their pathogenesis. The innate immune response also plays an indispensable role. The infiltration of monocytes and macrophages is always observed in the affected tissues of patients with autoimmune diseases (1). These cells stimulate and recruit other immune cells to diseased tissues by secreting pro-inflammatory cytokines and chemokines. Macrophages are important phagocytes acting against pathogens and serve as antigen-presenting cells that activate adaptive immune responses (2). Monocytes, macrophages, and adaptive T and B cells cooperatively contribute to chronic inflammation in autoimmune diseases.

The majority of systemic autoimmune diseases are multifactorial or polygenic; i.e., no single variant or gene can fully explain disease development. Genetic studies have revealed cumulative polygenic effects of numerous risk (or protective) variants with weak effect sizes on the susceptibility to developing autoimmune diseases. In the example of systemic lupus erythematosus (SLE), a prototypic autoimmune disease characterized by a broad spectrum of clinical symptoms and autoantibodies (3), patients in the highest polygenic risk score decile had a higher disease risk (odds ratio 30.3) compared with those in the lowest decile (4). In addition to polygenic risk factors, environmental factors are also critical in the development of autoimmune diseases. Concordance rates of 20–30% in monozygotic twins emphasize the importance of environmental factors in the development of autoimmune diseases (5). Transcriptome analysis of autoimmune diseases can capture dynamic genome-wide gene expression changes in immune cells reflecting both genetic and environmental stimulations.

In this review, we summarize how transcriptome analyses have improved our understanding of pathological immune cells, pathways, and genes in autoimmune diseases. We will introduce our peripheral blood transcriptome analyses on autoimmune diseases by the ImmuNexUT (Immune Cell Gene Expression Atlas from the University of Tokyo) consortium (6). We will also discuss the common experimental and analytical designs of transcriptome analyses and how transcriptome analysis can contribute to clinical decision-making for patient care.

## Blood Transcriptome and Identification of Interferon Signatures

Microarray studies, the pioneer transcriptome analyses conducted in autoimmune diseases, involve the hybridization of fluorescently labeled cDNA samples to probes on microarrays. Identification of the prominent interferon (IFN) genes involved in SLE by microarray analysis of blood mononuclear cells in patients and healthy controls in 2003 was an early hallmark discovery (7, 8) (**Table 1**). Using this differential gene expression approach, we compared the transcriptomes of diseased patients and a control group and statistically created a list of disease signature genes. Physiologically, plasmacytoid dendritic cells sense viral nucleic acids *via* TLR7 and TLR9 and produce type I IFN. Type I IFN increases the expression of major histocompatibility complex genes, induces chemokines and cytokines to recruit immune cells, and activates both innate and adaptive immune cells into an antiviral state (16). A recent genome-wide association study found that genetic variants in type I IFN gene clusters are associated with SLE risk (4). Elevation of the serum IFN-α concentration in blood cells is diagnostic of SLE (17). Several clinical trials and identification of the IFN signature led to the approval of anifrolumab, a human monoclonal antibody against IFNAR1 that significantly decreases the expression of type I IFN-induced genes, for the treatment of SLE in 2021 (18, 19). Enhanced expression of type I IFN signature genes in SLE is thus pivotal in the pathophysiology of SLE.

**Table 1:** Key immune cell transcriptome reports in SLE.

Authors	Reported year	Main experimental method	Main analytic method	Key findings	Reference
Bennett et al.	2003	Microarray of PBMC	DEG	IFN and granulopoiesis signature were elevated in SLE.	(7)
Baechler et al.	2003	Microarray of PBMC	DEG	IFN signature was elevated in SLE and it was related to more severe SLE.	(8)
Chaussabel et al.	2008	Microarray of PBMC	modular	IFN signatures and neutrophil signatures were correlated with SLE disease activity.	(9)
Lyons et al.	2010	Microarray of PBMC, CD4 and CD8 T cells, B cells, monocytes, and neutrophils	DEG, hierarchical clustering	Transcriptome differences observed in the PBMC largely reflected changes in their cellular composition. High IFN signatures in monocytes distinguished SLE from AAV and healthy controls.	(10)
McKinney et al.	2015	Microarray of CD4 and CD8 T cells	modular	Enhanced CD8 T-cell exhaustion and reduced CD4 T-cell co-stimulation signatures indicated a better prognosis in SLE and AAV patients.	(11)
Banchereau et al.	2016	Microarray of PBMC	modular	Plasmablast gene signature was the robust biomarker of disease activity. The neutrophil signature was correlated to active nephritis.	(12)
Arazi et al.	2019	single-cell RNA-seq of kidneys	graph-based clustering, trajectory analysis	IFN signatures were correlated between matched blood and kidney samples. Inflammatory blood monocytes gradually progressed to a phagocytic and then an M2-like macrophage. Naïve B cells differentiated to activated B cells with gradual elevation of ABC signature.	(13)
Nehar-Belaid et al.	2020	single-cell RNA-seq of PBMC	graph-based clustering	Subpopulations of major immune cells expressed high levels of IFN signatures. DN2 B cells were expanded in SLE.	(14)
Perez et al.	2022	single-cell RNA-seq of PBMC	Louvain clustering, modular, eQTL	Naïve CD4^+^ T cells are decreased and GZMH^+^CD8^+^ T cells are increased in SLE. Classical monocytes expressed the highest levels of IFN signature.	(15)

PBMC, peripheral blood mononuclear cells; IFN, interferon; DEG, differentially expressed genes; AAV, antineutrophil cytoplasmic antibody-associated vasculitis; ABC, age-associated B cell; eQTL, expression quantitative trait locus.

Peripheral blood IFN signature genes are expressed not only in SLE but also in other autoimmune diseases, including rheumatoid arthritis (RA), dermatomyositis, systemic sclerosis (SSc), and Sjögren’s syndrome (20–23). In RA, the most prevalent autoimmune disease, characterized by chronic autoimmune inflammation in the joints (24), the preclinical IFN signature predicts the development of arthritis (20). Moreover, a third to a half of established RA patients express IFN signature genes (21, 22). In a comparative study of five autoimmune diseases, the proportion of patients positive for type I IFN signature genes was 73% for SLE, 66% for dermatomyositis, 61% for polymyositis, 68% for SSc, and 33% for RA (22). These data suggest variable contributions of IFN signature genes to the pathogenesis of autoimmune diseases.

## Modular Analysis of the Blood Transcriptome and Cell-Type Gene Signatures

Analysis of differentially expressed genes at the transcriptome level is generally permissive to noise because of the large number of genes (usually > 10,000) analyzed and relatively limited sample numbers (usually ~100 and at most ~1,000). To overcome this “curse of dimensionality”, Chaussabel et al. applied modular analysis to peripheral blood mononuclear cell (PBMC) microarray data from 239 individuals and found two SLE disease activity-related transcriptional modules: IFN-signature and neutrophil genes (9). In modular analysis, sets of coordinately expressed genes are identified, and modules are constructed in a data-driven way. The modules typically represent functionally associated genes, such as plasma cell-type genes and IFN signature genes.

Banchereau et al. applied this modular analysis to data from the whole blood of 156 pediatric SLE patients and identified the plasmablast cell-type gene signature as the most robust biomarker of disease activity (12). The neutrophil module was activated in a subset of patients with active nephritis. The authors proposed a model of gradual disease progression, with early increases in IFN responses and B-cell differentiation into plasmablasts, followed by development of kidney disease and full-blown systemic inflammation fueled by myeloid cells, including neutrophils.

The most valuable lesson from these early transcriptome analyses is that blood transcriptomic differences are largely driven by compositional changes in immune cell populations. For example, blood transcriptome studies revealed that peripheral neutrophils are increased in lupus patients with active nephritis, but they could not determine the qualitative changes in neutrophils in detail.

## Bulk Immune Cell Transcriptome and Purified Cell-Type Specific Signatures

Lyons et al. isolated CD4 and CD8 T cells, B cells, monocytes, and neutrophils from patients with SLE or ANCA-associated vasculitis (AAV) and performed microarray analysis in comparison with microarray analysis of whole PBMCs (10). In their study, a substantial number of differentially expressed genes were identified only in the purified immune cells samples. Transcriptomic data from the monocytes differentiated AAV and SLE patients from each other and from controls more robustly compared with the PBMC transcriptomic data. Especially, IFN signature gene levels in monocytes distinguished the two diseases.

McKinney et al. analyzed the transcriptomes of purified CD8 and CD4 T cells using a module analysis approach (11). They found that enhanced CD8 T-cell exhaustion and reduced CD4 T-cell co-stimulation gene signatures indicated a better prognosis in SLE and AAV patients. In contrast, the CD8 T-cell exhaustion signature was associated with poor outcomes in patients with viral infections.

## ImmuNexUT; Bulk RNA-Seq Across Immune Cells and Immune-Mediated Diseases

**Figure 1:**
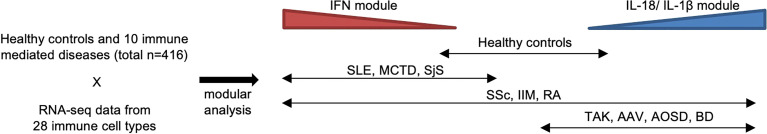
Heterogeneity of immune-mediated diseases in ImmuNexUT. In the ImmuNexUT flagship article, we applied weighted gene correlation network analysis (26) to immune cell gene expression data and systemically characterized the gene modules related to immune-mediated diseases. When we compared the expression of these modules between autoimmune disease patients and healthy controls, gene modules enriched with IFN-induced gene sets were overexpressed in autoimmune disease patients. SLE, mixed connective tissue disease (MCTD), Sjögren’s syndrome (SjS), systemic sclerosis (SSc), idiopathic inflammatory myopathy (IIM), and rheumatoid arthritis (RA). Gene modules enriched with IL-18 or IL-1β-induced gene sets were overexpressed in patients with autoinflammatory diseases: Behçet’s disease (BD) and adult-onset Still’s disease (AOSD). Takayasu arteritis (TAK) or ANCA-associated vasculitis (AAV) patients showed similar expression patterns to those in autoinflammatory disease patients. SSc, IIM, and RA patients were more heterogeneous compared with the other diseases.

Recently, we reported ImmuNexUT, a large database containing immune cell gene expression data from various immune-mediated diseases and many types of immune cells, in addition to healthy controls. In the first flagship study (6), we performed RNA sequencing (RNA-seq) in 28 types of purified bulk immune cells from healthy samples and 10 immune-mediated diseases (https://www.immunexut.org/). RNA-seq directly determines cDNA sequences using next-generation sequencers, independent of prior knowledge of genomic sequences, and the dynamic range to quantify gene expression levels is superior to that of microarrays (25). Modular analysis revealed that characteristically expressed gene modules in autoimmune diseases overlap IFN signature gene sets, and those in autoinflammatory diseases overlap IL-18- or IL-1β-activated gene sets (**Figure 1**). When comparing gene expression among individuals, we observed remarkable heterogeneity within diseases. Especially, patients with idiopathic inflammatory myopathy (IIM) with anti-MDA5 antibodies expressed high levels of IFN signature genes comparable with those in SLE patients, while the immune cell transcriptomes of the other IIM patients were heterogeneous. Anti-MDA5 antibodies in IIM are associated with life-threatening, rapidly progressing interstitial lung diseases (27). Because MDA5 is a cytosolic sensor of double-stranded RNA regulating IFN signaling, IFN signaling could have pathophysiological relevance, especially in this subtype of IIM.

## ImmuNexUT Sub-analysis of SSc

SSc is an intractable autoimmune disease characterized by skin and internal organ fibrosis and vasculopathy (28). The rate of disease-related mortality is higher in SSc patients than in other autoimmune disease patients (26). Treatment approaches such as immunosuppressive drugs, vasodilation, and antifibrotic therapy only partially ameliorate the disease. Bulk transcriptome analysis of the affected skin showed adaptive immune cell signatures were associated with early-phase disease, while fibroblast and macrophage cell type signatures were associated with advanced fibrosis (29). Valenzi et al. have performed a single-cell RNA-seq analysis of the affected lung tissue from SSc patients and have identified a large proliferating myofibroblast population (30). However, little was known about the transcriptomic changes of peripheral blood immune cells in SSc.

In a sub-analysis of SSc patients from ImmuNexUT, we performed a modular analysis of gene expression data from each immune cell type to compare SSc and healthy controls (31). Using a machine learning approach, random forest, we prioritized the most important gene co-expression module for discriminating SSc from healthy controls. An inflammatory gene module in CD16^+^ monocytes, including *KLF10*, *PLAUR*, *JUNB*, and *JUND* genes, showed the greatest capacity for discrimination. Integration with single-cell RNA-seq data from peripheral blood and interstitial lung disease lesional monocytes revealed a significant overlap of the gene module with a subgroup of monocytes. Because monocytes and their subpopulations are involved in tissue fibrosis (32–34), the pro-inflammatory monocyte subpopulation identified in that study might have a profibrotic capacity as well.

In addition, in a subsequent analysis comparing the transcriptomes of early-stage SSc (disease duration < 5 years) and late phase SSc (35), we revealed distinct differentially expressed genes in regulatory T cells (Tregs). In an integrative analysis with single-cell RNA-seq data, we performed deconvolution (36), a method to statistically estimate the proportions of cell subpopulations based on mixed cell data. Again, we found expansion of an activated subpopulation of Tregs in early-stage SSc (and also in IIM) with marked differences in gene expression. The role of Tregs in fibrosis is controversial because whether Tregs are profibrotic or antifibrotic depends on the experimental animal model (37). Our data suggest a profibrotic role of Tregs during the early phase of fibrotic autoimmune diseases.

In sharp contrast, a similar analysis of ImmuNexUT data in AAV patients versus healthy controls identified upregulation of a gene module related to neutrophil extracellular trap formation (NETosis) in AAV patients (38), revealing the importance of NETosis in the pathogenesis of AAV. In fact, the neutrophil enzymes MPO and PR3 are released by NETosis and are targets of autoantibodies in AAV (39, 40).

Because these analyses are performed using a data-driven hypothesis-free approach, the identified candidate immune cell populations, pro-inflammatory monocytes, and activated Tregs, could be characteristic of SSc and worth further investigation. These results also taught us that even the gene expression data from highly purified bulk immune cells in ImmuNexUT are influenced by changes in the sizes of target immune cell subpopulations.

## Single-Cell RNA-Seq of Blood Immune Cells in SLE

The complex immune system involves interactions among multiple types of immune cells. Advances in single-cell RNA-seq technology have allowed comprehensive identification and characterization of distinct immune cell subpopulations at a single-cell resolution (41–44). However, currently available single-cell RNA-seq technologies capture only a few thousand of the most highly expressed genes per cell, resulting in a sparse gene expression picture with higher technical noise compared with bulk-cell RNA-seq technologies.

Nehar-Belaid et al. reported large-scale single-cell RNA-seq profiles (> 350,000 single cells) in the PBMCs of child and adult SLE patients (14), comprising 33 child and 11 adult patients. That study revealed that a small subpopulation of monocytes and other major immune cells expressing high levels of IFN signature genes was expanded and capable of distinguishing SLE from healthy controls. The results imply that enhanced expression of IFN signature genes in peripheral blood in many autoimmune diseases is, in fact, derived from a small subpopulation of high IFN-expressing cells. They also report an expansion of the B cell subpopulation expressing both IFN signatures and extrafollicular, potentially autoreactive, double negative switched memory B cell phenotype (CD27^-^IgD^-^CXCR5^-^CD11c^+^ DN2 cells) (45). This B cell subpopulation was enriched with monogenic lupus-associated genes, which suggest a causal role of this subpopulation in the SLE. DN2s are closely related to age-associated B cells (ABC), sharing their surface marker CD11c (45). ABCs are implicated in both aging and autoimmunity. In addition, a fraction of SLE CD8^+^ T cells showed upregulation of cytotoxic genes.

Recently, Perez et al. also reported larger-scale single-cell RNA-seq profiles (>1.2 million single cells) in the PBMC of 162 SLE patients and healthy controls both from European and Asian ancestries (15). They revealed a reduction in naïve CD4^+^ T cells and an increase of GZMH^+^CD8^+^ T cells in SLE. GZMH^+^CD8^+^ T cells expressed cytotoxic, exhaustion, and IFN signatures. GZMH^+^CD8^+^ T cells were clonally expanded and restricted, and they may have a pathogenic role in the disease process. The presence of an atypical B cell population, sharing some ABC markers (CD11c^+^TBX21^+^) was confirmed. Classical monocytes expressed the highest levels of IFN signature. These results support the utility of single-cell RNA-seq technology to identify and characterize disease-relevant subpopulations of immune cells.

## Single-Cell RNA-Seq Analysis of Tissue and Proinflammatory Macrophages

Peripheral blood immune cells are popular cell models for transcriptome analyses of autoimmune diseases, probably because of the better access to such cell samples in comparison with immune cells infiltrating affected organs, such as the synovium in RA. However, whether peripheral blood immune cells accurately capture the immunological abnormalities of the affected organs is debated.

In a single-cell profiling experiment of immune cells, Wu et al. simultaneously analyzed peripheral blood and synovial CD45^+^ mononuclear cells from RA patients with and without anti-citrullinated peptide antibodies (ACPAs), hallmark autoantibodies of RA (46). Peripheral blood analysis revealed important characteristics of RA, such as an expansion of cytotoxic CD4 T cell population (47, 48), while synovial gene profiling revealed correlates of ACPA status, including up-regulation of *CCL13*, *CCL18*, and *MMP3* in myeloid cell subsets of ACPA-negative RA compared with ACPA-positive RA. Although reports directly comparing the utility of single-cell RNA-seq analysis in blood versus synovium are scarce, synovial immune cell profiling seems more sensitive than peripheral blood profiling in the detection of immunological correlates of RA subpopulations.

As part of the Accelerating Medicines Partnership consortium, Zhang et al. profiled 51 synovial tissue samples from both RA and osteoarthritis patients by combining single-cell RNA-seq, bulk RNA-seq, and mass cytometry data (49). They found expansion of *IL1B* ^+^ pro-inflammatory monocytes, *ITGAX* ^+^ *TBX21* ^+^ autoimmune-associated B cells, *PDCD1* ^+^ peripheral helper T cells (Tph), and follicular helper T cells (Tfh) in the RA synovium. Tph cells are a new subset of helper T cells previously identified in the RA synovium, which can induce plasma cell differentiation *in vitro* (50). In contrast to classical CXCR5^+^ Tfh cells, Tph cells lack surface CXCR5 and produce CXCL13 and IL-21 to recruit both Tfh and B cells. Therefore, Tph cells appear to have an important role in the local autoantibody production in the inflamed tissues.

Kuo et al. focused on synovial macrophages and, using single-cell RNA-seq, identified HBEGF^+^ pro-inflammatory macrophages enriched in the RA synovium (51). This macrophage subpopulation has the capacity to promote fibroblast invasiveness in an EGF receptor-dependent manner. Also, synovial fibroblasts can promote the HBEGF+ inflammatory phenotype of macrophages. The crosstalk between pro-inflammatory macrophages and synovial fibroblasts might be important in the pathophysiology of chronic inflammation in RA joints. Interestingly, synovial pro-inflammatory macrophages express some common gene markers, such as *PLAUR, NR4A2*, and *CXCL2*, to those expressed in inflammatory monocytes that we identified in the peripheral blood of patients with SSc (31). This may imply similarities between monocytes and macrophages in the pathophysiology of RA or SSc. Zhang et al. performed integrative single-cell RNA-seq analysis of > 300,000 cells in bronchoalveolar lavage samples from COVID-19, RA, and other inflammatory diseases, all of which exhibited expansion of CXCL10^+^CCL2^+^ inflammatory macrophages, experimentally driven by a combination of the pro-inflammatory cytokines IFN-γ and tumor necrosis factor (TNF)-α (52). That study is a good example demonstrating the utility of cross-disease data analysis to reveal shared disease processes, in this case, the expansion of inflammatory macrophage subpopulations in various disease conditions.

## Single Cell RNA-Seq Analysis of Lupus Nephritis

In the Accelerating Medicines Partnership consortium, Arazi et al. reported immune cell clusters in lupus nephritis samples (13). Trajectory analysis, based on the similarity of single-cell gene expression, suggested a gradual progression of inflammatory blood monocytes to a phagocytic and then an alternatively activated (M2-like) macrophage phenotype in the kidney. Alternatively, activated (M2) macrophages have an anti-inflammatory function and regulate wound healing (53). Trajectory analysis also revealed a gradual differentiation from naïve B cells to activated B cells with an incremental elevation of the ABC gene signature. They compared the CD8^+^ T cell exhaustion signatures, previously reported to be associated with lower lupus flares (11), between blood and kidney. The CD8^+^ T cell exhaustion signature of patients with lupus nephritis was high in blood but not in the kidney. In that report, IFN signature gene expression was correlated between matched blood and kidney samples (n = 10), implying that the IFN response may be an extrarenal process. When comparing urine and kidney leukocytes, the urine samples had a higher frequency of phagocytic macrophages, and high transcriptomic correlations were observed between the samples. Urine cells may serve, at least partially, as an alternative to their kidney counterparts. Der et al. reported tubular and keratinocyte single-cell RNA-seq data (54). They showed that the IFN signatures in tubular cells and keratinocytes distinguished patients with lupus nephritis from healthy control subjects. Moreover, high IFN and fibrotic gene signatures in tubular cells were associated with failure to respond to treatment.

## Dynamic eQTL Analysis of Autoimmune Diseases

Genome-wide association studies (GWASs) have identified tens of thousands of gene variants significantly associated with diseases (55). In RA and SLE, large-scale GWASs have identified more than a hundred robust genetic variants associated with each disease (4, 56, 57). However, most of the identified causal genetic variants of these autoimmune diseases are located in non-coding enhancer regions of the genome (58, 59), and their biological mechanisms in autoimmunity are not self-explanatory.

**Figure 2:**
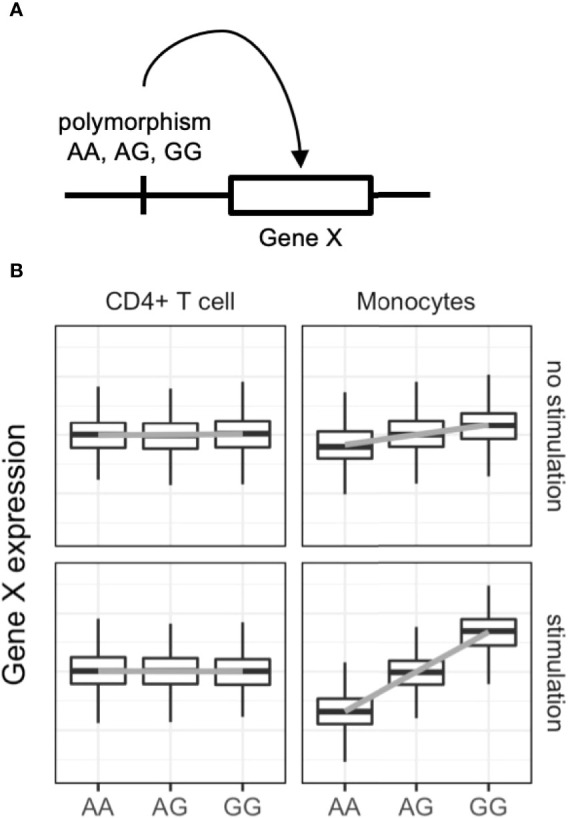
Dynamic eQTL of the immune cells. **(A)** Schematic representation of expression quantitative trait locus (eQTL) analysis. eQTL is an association test between common single nucleotide polymorphisms and nearby (in most cases) gene expression. **(B)** An example of dynamic eQTL in immune cells. eQTL effect sizes, expressed as standardized linear regression coefficients, are cell-type and cell-state dependent. In this example stimulated monocytes have four times more eQTL effects compared with unstimulated monocytes.

Expression quantitative trait locus (eQTL) analysis evaluates associations between genetic variants and gene expression levels (**Figure 2**) *via* linear regression of normalized gene expression levels and the tested allele dosages. Genetic variants associated with diseases have greater eQTL effects compared with other variants (60). The variants can alter the binding capacity of transcription factors to the enhancers or promotors of genes and thus regulate the expression of nearby genes (61). Importantly, eQTL analysis provides directional information on the effects of the tested single nucleotide polymorphisms (SNPs) on target gene expression. In the example in **Figure 2**, if the G allele of gene X is a GWAS risk SNP, enhanced expression of gene X, especially in stimulated monocytes, is a candidate biological mechanism. The following are limitations of eQTL analysis: large-scale transcriptomic data with typically more than 50–100 samples are required (62); eQTL effects are variable among immune cell types (63, 64) and stimulations (65, 66); overlap of GWAS and eQTL signals do not guarantee causality of the identified genes (67); and only common SNPs (allele frequency > 0.01–0.05) can be tested, while rare variants cannot.

ImmuNexUT includes samples from more than 400 individuals comprising both healthy controls and immune-mediated disease patients (unstimulated and stimulated immune cells *in vivo*) and 28 immune cell types (**Figure 1**). Using eQTL analysis of ImmuNexUT data (6), we identified immune cell-type specific and disease-specific eQTLs. A median of 7,092 genes was significantly regulated by eQTL in each immune cell type. Partitioning of GWAS heritability with cell-type-specific eQTL data revealed candidate causal immune cells in autoimmune diseases: effector Tregs in RA and naïve and unswitched memory B cells in SLE. Colocalization analysis between SLE GWAS and ImmuNexUT eQTL data identified candidate causal genes. For example, decreased expression of *ARHGAP31* in plasmablasts was associated with SLE. Those results highlight the power of eQTL analysis in elucidating autoimmune disease mechanisms.

In the OneK1K cohort, Yazar et al. collected PBMC single-cell RNA seq data of 1.27 million cells from 982 healthy donors (68). They identified 14 major immune cell populations and eQTL analysis identified variable number of independent eQTLs (399 in plasma cells and 6473 in naïve and central memory CD4^+^ T cells). They showed the overlap of various autoimmune GWAS loci and immune cell eQTLs. Also, using single-cell RNA-seq data of SLE patients, Perez et al. performed eQTL analysis in the eight most abundant cell types (15). Their analysis identified 3331 genes with at least one eQTL in a cell type. The advantage of these single-cell RNA-seq based approaches over a bulk RNA-seq based approach is the ability to compute dynamic transcriptional transitions of cellular state using pseudotime analysis. However, cell-type-specific effects of the population with a small fraction size could not be estimated because of the limited number of cells. For example, the cell-type-specific eQTL effects of Treg were not examined in these single-cell RNA-seq eQTL studies, despite the importance of Treg in its ability to regulate autoimmune reactions. Further, Yazar et al. detected fewer eQTL signals in each immune cell type despite the higher number of analyzed individuals (n=982) in comparison to the ImmuNexUT project (n=416). Therefore, the bulk RNA-seq based approach has some advantages in the detection power of immune cell eQTL analysis over the single-cell RNA-seq based approach.

## Clinical Application of Transcriptome Analyses

In clinical trials of anifrolumab for SLE, higher baseline levels of IFN signature genes were associated with a better clinical response (18, 19). In those reports, IFN gene signatures, classified as either high or low, were estimated by whole-blood quantitative PCR-based analysis of four genes (*IFI27*, *IFI44*, *IFI44L*, and *RSAD2*). These results are consistent with the specific binding of anifrolumab to INFAR1. Similarly, baseline protein levels of TNF and soluble interleukin-6 (IL-6) receptor are associated with the response to their respective targeted treatment in RA patients (69, 70). In addition, the B cell subpopulation levels are associated with the treatment responses of B-cell-targeted anti-CD20 rituximab therapy in AAV or SLE patients (71, 72). As seen in these examples, gene or protein expression levels or cell populations appear to be promising candidate biomarkers for predicting treatment responses. However, these biomarkers do not have enough sensitivity or specificity for routine clinical use. One reason might be the use of blood for estimating the biological activity of cytokines because they typically act locally *via* both autocrine and paracrine manners on other cells (73).

Interestingly, peripheral blood levels of IFN signature genes are associated with the response to RA treatment. For example, high IFN signature gene levels are associated with better responses to treatments with a TNF-α inhibitor (74), the IL-6 receptor inhibitor tocilizumab (75), and the T-cell-blocking CTLA4-Ig abatacept (76). Meanwhile, IFN signature gene levels were associated with non-responsiveness to methotrexate (77) and B-cell-targeting rituximab (78, 79). Those reports imply that the IFN signature status could be used for immunological stratification of RA patients, thereby affecting treatment choice.

Transcriptome analysis of blood samples can capture both cytokine signaling gene expression (e.g., IFN signature) and immune cell-type signature gene expression (e.g., plasmablast signature). Therefore, peripheral blood transcriptome profiling is a candidate approach in precision medicine based on biologically targeted therapies. In addition, the peripheral blood transcriptome can be used to predict the natural history of various autoimmune diseases. In the report by McKinney et al., the “exhaustion signature” of purified CD8^+^ T cells was associated with a lower number of disease flares in SLE and AAV patients (11). Serial peripheral blood transcriptome analyses have also identified gene signatures preceding RA flares, associated with B cell activation and subsequent expansion of CD45^-^CD31^-^PDPN^+^ pre-inflammatory mesenchymal cells, which show gene expression features similar to those of inflammatory synovial fibroblasts (80). With the establishment of accurate and robust prognostic prediction by transcriptome analysis, treatments can be tailored based on the obtained transcriptomic data.

In RA, several studies have shown that gene expression analysis of joint synovial tissues can identify disease subtypes and predict treatment responses (81–84). Humby et al. combined gene expression with immunohistological analyses of the synovium in treatment-naïve early-stage RA patients (82). Three groups of patients were identified according to the synovial tissue phenotype: pauci-immune fibroid, diffuse-myeloid, and lympho-myeloid patients. Elevation of myeloid- and lymphoid-associated gene expression was strongly correlated with disease activity and the treatment (90% methotrexate) response at six months. Lewis et al. also profiled early RA-associated genes using RNA-seq and showed that the gene signature of synovial plasma cells predicts future joint damage (83). In addition, they compared the blood and synovial RNA-seq data and showed that synovial genes are more differentially expressed among the different synovial tissue phenotypes. In addition, the blood IFN signature was associated with synovial B and plasma cell infiltration, which may at least partially explain the correlation between the blood IFN signature and treatment effects (78). Finally, Humby et al. reported a clinical trial comparing the effects of the anti-CD20 monoclonal antibody rituximab and anti-IL6 receptor tocilizumab in non-responders to anti-TNF therapy (84). Baseline synovial biopsies were subjected to classical histological and RNA-seq analyses to classify the samples as B-cell rich or poor. In B-cell-poor RA, tocilizumab treatment had a better response rate than that of B-cell targeted rituximab therapy. An important finding of that study was that RNA-seq-based classification was better correlated to the treatment response than was histological classification. Currently, synovial biopsies are not standard clinical practice for RA, but they might become such, considering the limited invasiveness of the biopsy procedure and potential benefits of precision medicine based on the molecular disease subtype.

## Conclusion

Here, we reviewed how the transcriptome analysis of immune cells has improved our understanding of the pathophysiology of autoimmune diseases with a focus on the ImmuNexUT analysis that we recently reported. Single-cell RNA-seq analyses have been expanding our knowledge of the immune cells infiltrating disease lesions. Inflammatory monocytes and macrophages are expanded across various autoimmune and inflammatory diseases. IFN pathway, neutrophils, and B cell subpopulations have been robust signatures in SLE in different experimental and analytic methods (**Table 1**). These cell populations could have pathophysiological relevance to autoimmune diseases. Expression QTL analysis, integrated with GWAS data, has identified candidate causal immune cells and genes. Genes identified by transcriptome analyses of the peripheral blood or the RA synovium can be used as clinical predictive or prognostic biomarkers. Therefore, transcriptome analysis of autoimmune diseases is not only a useful tool for the investigation of disease mechanisms but also has the potential for direct clinical application in future precision medicine.

## Author Contributions

YN, HY, and KF conceived and designed the study concept. YN wrote the original manuscript draft. HY and KF reviewed and edited the manuscript. All authors contributed to the article and approved the submitted version.

## Funding

This work was supported by JSPS KAKENHI Grant Number 19K16973, GSK Japan Research Grant 2020, and the Social Cooperation Program, Department of Functional Genomics and Immunological Diseases, supported by Chugai Pharmaceutical. The funders were not involved in the study design, collection, analysis, interpretation of data, the writing of this article or the decision to submit it for publication.

## Conflict of Interest

YN belongs to the Social Cooperation Program, Department of Functional Genomics and Immunological Diseases, supported by Chugai Pharmaceutical, and has received financial support and/or speaking fees from BMS, Chugai, GlaxoSmithKline, Kissei, Mitsubishi Tanabe, and Pfizer. KF has received speaking fees and/or honoraria from Tanabe Mitsubishi, Bristol Myers, Eli Lilly, Chugai, Jansen, Pfizer, Ono, AbbVie, Ayumi, Astellas, Sanofi, Novartis, Daiichi Sankyo, Eisai, Asahi Kasei, Japan Blood Products Organization, and Kowa, and has received research grants from Tanabe Mitsubishi, Bristol Myers, Eli Lilly, Chugai, AbbVie, Ayumi, Astellas, Sanofi, Eisai, Tsumura and Co., and Asahi Kasei. The remaining author declares that the research was conducted in the absence of any commercial or financial relationships that could be construed as a potential conflict of interest.

## Publisher’s Note

All claims expressed in this article are solely those of the authors and do not necessarily represent those of their affiliated organizations, or those of the publisher, the editors and the reviewers. Any product that may be evaluated in this article, or claim that may be made by its manufacturer, is not guaranteed or endorsed by the publisher.
